# Epidemiology, changing resistance trends and serotypes of *Streptococcus pneumoniae* in children in Chongqing, China, 2019–2024: a multicenter study

**DOI:** 10.3389/fcimb.2025.1700449

**Published:** 2026-01-05

**Authors:** Jie Zhao, Chunmei Jing, Xiaoyan Yu, Zhongzheng Xiong, Yupei Xiang, Fang Liu, Xiaoqiang Li

**Affiliations:** 1 Department of Clinical Laboratory Children’s Hospital of Chongqing Medical University, National Clinical Research Center for Children and Adolescents' Health and Diseases, Ministry of Education Key Laboratory of Child Development and Disorders, Chongqing Key Laboratory of Child Rare Diseases in Infection and Immunity, Chongqing, China; 2 Department of Clinical Laboratory, Dian Jiang Peoples Hospital of Chongqing, Chongqing, China; 3 Department of Clinical Laboratory,Chongqing Jiulongpo District Science City People's Hospital, Chongqing, China; 4 Department of Clinical Laboratory, Chongqing Red Cross Hospital (People’s Hospital of Jiangbei District), Chongqing, China

**Keywords:** children, epidemiology, resistance rate, serotype, *Streptococcus pneumoniae*, vaccine

## Abstract

**Objective:**

To investigate the clinical characteristics, temporal trends in antimicrobial resistance, and distribution of bacterial serotypes of *Streptococcus pneumoniae* (*S. pneumoniae*)in children in Chongqing region from 2019 to 2024.

**Methods:**

*S. pneumoniae* isolates and corresponding epidemiological data were collected from multi-center laboratories. Antimicrobial susceptibility testing was performed in the central research laboratory for each study period from 2019 to 2024*, and* the results were interpreted according to the breakpoint criteria specified in the Clinical and Laboratory Standards Institute (CLSI) M100-S34 guidelines (2024 edition). Capsular serotyping of *S. pneumoniae* was performed using the capsular swelling test, and vaccine coverage rate were calculated.

**Results:**

A total of 17,180 *S. pneumoniae* isolates were isolated over six years, accounting for 17.2% of all clinically isolated pathogenic bacteria and 45.9% of all Gram-positive bacteria. The isolates were mainly obtained from respiratory tract specimens (97.9%), followed by blood specimens (1.1%). *S. pneumoniae* was predominantly isolated from preschool, toddler and Infants, with isolation rates of 32.5%, 30.5% and 25.9%, respectively, together accounting for 88.8% of all *S. pneumoniae* isolates. The detection rates of penicillin-susceptible *S. pneumoniae* (PSSP), penicillin-intermediate *S. pneumoniae* (PISP), and penicillin-resistant *S. pneumoniae* (PRSP) were 83.7% (14196/16964), 14.8% (2513/16964), and 1.5% (255/16964), respectively. During the study period, the resistance rate to penicillin, trimethoprim/sulfamethoxazole, erythromycin, clindamycin, cefotaxime*, and* cefepime presented a significant downward trend. Except for vancomycin and linezolid. the resistance rates to all tested drugs in the PRSP group were higher than those in the PSSP group. Among different age groups, the resistance rate to trimethoprim/sulfamethoxazole and clindamycin were highest in toddler stage children, whereas erythromycin resistance was highest in preschool children. The resistance rates to penicillin, chloramphenicol, cefotaxime, and cefepime also differed significantly across age groups. The resistance rates to trimethoprim/sulfamethoxazole, levofloxacin, and moxifloxacin were higher in non-IPD group than in IPD group, whereas chloramphenicol resistance was lower. The average annual detection of *S. pneumoniae* decreased in post-COVID-19, and, except for chloramphenicol, resistance rates to all other antibacterial drugs were lower than those in the pre-COVID-19 period. Thirteen serotypes were identified, except for 8 (1.3%) non-typeable isolates. The top five serotypes, 19F (n = 207, 34.5%),14(n = 65, 10.9%),19A (n = 61, 10.2%), 6B (n = 59, 9.8%) and 1 (n = 52, 8.7%), accounted for 74.1% of all isolates. PCV7, PCV10, and PCV13 covered 388 (64.6%), 440 (73.3%), and 501 (83.5%) strains, respectively.

**Conclusion:**

The resistance rates of *S. pneumoniae* to penicillin, trimethoprim/sulfamethoxazole, erythromycin, clindamycin, cefotaxime, and cefepime show significant downward trends over the six-year study period. The pneumococcal conjugate vaccine PCV13 can effectively cover the major drug-resistant serotypes in China, and PCV 13 is therefore recommended for the prevention of *S. pneumoniae* infection. These findings contribute to informed and clinical policy decisions for prevention and treatment.

## Introduction

Infections caused by *S. pneumoniae* constitute a significant threat to human health (Narciso et al., 2025). It can cause not only noninvasive pneumococcal diseases (NIPDs), such as pneumonia, conjunctivitis, sinusitis, otitis media and bronchitis, but also severe invasive pneumococcal diseases (IPDs), including pleurisy, meningitis and sepsis, especially in children and the elderly (Zainel et al., 2021; Li et al., 2023; Zeng et al., 2023; Narciso et al., 2025). According to the World Health Organization (WHO), *S. pneumoniae* is the most common pathogen causing pneumonia. More than 800,000 children die from pneumococcal diseases annually, particularly in developing and underdeveloped countries (Johnson et al., 2010). Based on the 2015 Global Burden of Diseases (GBD) data, approximately 64% of pneumonia deaths in children under 5 years of age were due to bacterial infections (Mai et al., 2023).

Multivalent pneumococcal vaccine can significantly reduce the incidence of diseases caused by *S. pneumoniae*. At present, more than 100 pneumococcal serotypes have been identified worldwide (Ganaie et al., 2020; Pimenta et al., 2021). However, only some of these serotypes would cause pneumococcal disease (Namkoong et al., 2016). Some countries have included pneumococcal conjugate vaccines (PCVs) in their national immunization programs, and the use of vaccines targeting specific serotypes has significantly reduced IPDs caused by the vaccine serotypes (VTs) (Chan et al., 2019). Vaccines such as PCV7,PCV10 and PCV13 are currently available in China. However, none of these vaccines has yet been included in the national immunization program, and their relatively high cost has resulted in low vaccination coverage (Men et al., 2020; Wang et al., 2021). The use of PCVs can lead to changes in the serotype distribution of *S.pneumoniae*. Although these vaccines provide protection against VTs, they have also been associated with an increase in non-vaccine serotypes (NVTs) (Lo et al., 2022). This increase in NVTs is of particular concern, especially as these serotypes are increasingly resistant to antibiotics commonly used to treat pneumococcal disease (Zhao et al., 2020).

According to existing literature reports, the serotype distribution of *S. pneumoniae* varies by time, region and population (Narciso et al., 2025). The WHO recommends pneumococcal vaccination to prevent pneumococcal infection, and vaccine selection should be guided by local serotype distribution to achieve optimal effectiveness. Therefore, understanding the distribution of pneumococcal serotypes in this region has crucial clinical value. Although epidemiological surveillance reports on *S. pneumoniae* are published annually, differences in prescribing practices across regions may lead to distinct antimicrobial resistance of this pathogen (Zhou et al., 2022; Tran-Quang et al., 2023; Kawaguchiya et al., 2024; Lyu et al., 2024). However, multicenter studies on changes in antimicrobial resistance and serotypes distribution of *S. pneumoniae* among children in the Chongqing area have not yet been reported.

To fill this gap, we conducted a 6-year retrospective multicenter study from 2019 to 2024, analyzing 17,180 clinical isolates of *S. pneumoniae* from children to characterize their clinical features and temporal trends in antimicrobial resistance. Serotype distribution and estimated vaccine coverage were also assessed to provide an evidence base for the effective prevention and treatment of *S. pneumoniae* infections in children, for reducing the emergence of multidrug-resistant strains, and for informing vaccine selection.

## Materials and method

### Patients and bacteria enrollment

Between January 2019 and December 2024, four hospitals within the Southwest China Pediatric Laboratory Specialty Alliance participated in this study. These hospitals included the Children’s Hospital of Chongqing Medical University, Dian Jiang People’s Hospital of Chongqing, Chongqing Jiulongpo District Science City People’s Hospital, and Chongqing Red Cross Hospital.

Patients younger than 18 years with clinically diagnosed community acquired respiratory tract infections (CARTIs), such as community-acquired pneumonia, acute exacerbation of chronic bronchitis, acute and/or chronic pharyngitis, tonsillitis or sinusitis occurring in the community or within 48 hours after hospital admission, were included. Patients older than 18 years, as well as nonpathogenic strains, duplicate isolates, bacteria colonization without clinical evidence of infection, and non-community-acquired pathogenic strains isolated from patients who had been hospitalized for more than 48 hours, were excluded from the study. All medical institutions involved in this multicenter study adopted unified inclusion and exclusion criteria.

### Isolation and identification of strains

Strain identification was performed by each subcenter. During the six-year research period, the strain identification systems and reagents did not change at any subcenter. All research centers adopted the same protocol for bacterial identification and internal quality control. Moreover, all clinical laboratories simultaneously participated in and passed inter-laboratory quality assessments organized by the national and Chongqing Clinical Laboratory Centers, ensuring the accuracy and consistency of identification and antimicrobial susceptibility test results.

### Quality control in strain identification

Strains isolated from specimens obtained by sterile site puncture (e.g., blood, CSF) were considered pathogenic. The majority of noninvasive strains were cultured from sputum, and the quality of sputum specimens was assessed to determine whether the isolates were pathogenic. In some cases, imaging evidence (such as chest X-ray) was also used to determine the pathogenicity of cultured strains. Furthermore, based on combined results of culture and immunopathological examinations, isolates were classified as pathogenic or colonizing bacteria, and only pathogenic isolates were included in the study. Specimens from nasopharyngeal swabs and sputum samples with more than 25 white blood cells (WBCs) and fewer than 10 squamous epithelial cells per low-power field were considered qualified. Other specimens, such as blood, pleural effusion, and cerebrospinal fluid (CSF), were considered sources of invasive bacterial infection.

Specimens were collected by specialized sampling personnel or physicians, and strains were isolated on Columbia agar supplemented with 5% sheep blood (BD Medical Technology, NJ, USA), which were incubated at 35 °C for 24–48 hours in an atmosphere containing 5% carbon dioxide (CO_2_). All isolates were identified based on typical colony morphology and optochin susceptibility, and the results were confirmed by matrix-assisted laser desorption/ionization time-of-flight mass spectrometry (MALDI-TOF MS; Vitek MS system; BioMerieux, Rhône, France).

### Antimicrobial susceptibility testing

**Antimicrobial susceptibility testing** (AST) was performed for all 17180 confirmed *S. pneumoniae* isolates using the VITEK 2 Compact system (BioMerieux, France) with the AST-GP68 card, the TDR-J100 System(STR-96), and the Etest method. All tests were conducted strictly in accordance with the guidelines of the Clinical and Laboratory Standards Institute (CLSI) (2024). The following commonly used antimicrobials were tested: penicillin, trimethoprim/sulfamethoxazole, levofloxacin, moxifloxacin, vancomycin, linezolid, erythromycin, clindamycin, chloramphenicol, ceftriaxone, cefotaxime, and cefepime. Etest strips (Wenzhou Kangtai Company, Wenzhou, China) were used to determine minimum inhibitory concentrations (MICs) when required. AST results were interpreted according to the Clinical and Laboratory Standards Institute (CLSI) M100-S34 guidelines (2024) (Clinical and Laboratory Standards Institute (CLSI), 2024).*S. pneumoniae* ATCC 49619 was used as the quality control strain and was included in each batch of tests to ensure accuracy. The oral penicillin breakpoints were used to classify isolates as penicillin-susceptible (MIC ≤0.06 μg/mL), penicillin-intermediate (MIC 0.12-1 μg/mL), and penicillin-resistant (MIC ≥2 μg/mL). Penicillin non-meningitis breakpoints (susceptible, MIC ≤2 μg/mL; resistant, MIC ≥8 μg/mL) and penicillin meningitis breakpoints (susceptible, MIC ≤0.06 μg/mL; resistant, MIC ≥0.12 μg/mL) were also applied to evaluate susceptibility. For ceftriaxone, the non-meningitis breakpoints (susceptible, MIC ≤1 μg/mL; resistant, MIC ≥4 μg/mL) and meningitis breakpoints (susceptible, MIC ≤0.5 μg/mL; resistant, MIC ≥2 μg/mL) were used to classify isolates as susceptible and resistant (Clinical and Laboratory Standards Institute (CLSI), 2024).

### Serotyping and vaccine coverage

A total of 600 *S. pneumoniae* isolates were serotyped using the capsular swelling test. Each year from 2019 to 2024, 100 isolates were randomly selected (25 isolates per subcenter), with three isolates per center obtained in January, and two isolates per month from February to December. Capsular type-specific antisera (Statens Serum Institute, Copenhagen, Denmark) were used for the capsular swelling test, which was performed strictly in accordance with the manufacturer’s instructions. The typing antisera allowed determination of serotypes covered by the 23-valent polysaccharide vaccine (1, 2, 3, 4, 5, 6B, 7F, 8, 9N, 9V, 10A, 11A, 12F, 14, 15B, 17F, 18C, 19A, 19F, 20, 22F, 23F and 33F), as well as 6A. Other serotypes were classified as non-typeable. The 7-valent vaccine (PCV7, covering serotypes 4, 6B, 9V, 14, 18C, 19F and 23F), the 10-valent vaccine (PCV10, additionally covering serotypes 1, 5 and 7F on the basis of PCV7) and 13-valent vaccine (PCV13, additionally covering serotypes 3, 6A and 19A compared with PCV10) were considered in the calculation of vaccine coverage. Based on the distribution of *S. pneumoniae* serotypes, the proportion of isolates with serotypes included in each vaccine was calculated as the vaccine coverage rate.

### Definitions

#### Age groups of children

The age groups for children were defined according to the 4th edition of *Child Health Care* as follows: neonates,≤28 days; infancy, 29 days to <1 year; toddler stage, 1 to <3 years; preschool stage, 3 to <6 years; school age, 6 to <12 years; adolescence, 12 to <18 years.

IPD and non-IPD isolates

Isolates obtained from sterile sites (such as cerebrospinal fluid, blood, and pleural fluid)were defined as IPD strains, and all other isolates were defined as non-IPD strains.

### COVID-19 period division

The study period (2019–2024) was divided into three intervals: the pre-COVID-19 period (2019), the COVID-19 pandemic period (2020–2022), and the post-COVID-19 period (2023–2024)

### Statistical analysis

Raw data were processed using WhONET 5.6 software and analyzed with GraphPad Prism 5. Differences in age were further assessed using the Mann-Whitney U test, and categorical data were evaluated using the chi-square test or Fisher’s exact test. Statistical significance was defined as a two-tailed P value < 0.050.

## Results

### Population characteristics

A total of 17180 *S. pneumoniae* isolates were collected from children: 3283 in 2019, 1814 in 2020, 3437 in 2021, 2903 in 2022, 3416 in 2023, and 2327 in 2024. The isolates were mainly obtained from children in the toddler stage(1–3 years), accounting for 30.5%, and the preschool stage (3–6 years), accounting for 32.5%. From 2019 to 2024, the detection rates of *S. pneumoniae* differed significantly among age groups (P<0.0001). In terms of sex, 56.7% of the 17180 participants were male. There was no significant difference in the overall detection rate of *S. pneumoniae* between sexes, except in 2019, 2020 and 2021 (P<0.050). With respect to seasonal distribution, *S. pneumoniae* was detected throughout the year. The detection rates of *S. pneumoniae* in spring (March to May), summer (June to August), and autumn (September to November) and winter (December to February) were 24.7%, 21.5%, 28.6%, and 25.1%, respectively, with higher detection rates in autumn and winter. In 2020, especially at the beginning of the year, the number of pediatric patients was significantly lower due to the initial outbreak of the COVID-19 pandemic,. The most common clinical diagnosis was pneumonia, accounting for 62.8% of cases (**Table 1**).

**Table 1:** Characteristics of the study population.

Characteristics	Total	Year, n (%)					
		2019	2020	2021	2022	2023	2024
Participants Age group (years)	17180	3283	1814	3437	2903	3416	2327
Neonates (≤28d)	37 (0.2)	7 (0.2)	7 (0.4)	10 (0.3)	3 (0.1)	7 (0.2)	3 (0.1)
Infancy (29d-1y)	4360 (25.9)	1060 (32.3)	664 (36.6)	937 (27.3)	605 (20.8)	626 (18.3)	468 (20.1)
Toddler stage (1-3y)	5234 (30.5)	1132 (34.5)	598 (33.0)	1127 (32.8)	825 (28.4)	892 (26.1)	660 (28.4)
Preschool stage (3-6y)	5659 (32.5)	877 (26.7)	449 (24.8)	1129 (32.8)	1088 (37.5)	1292 (37.8)	824 (35.4)
School age (6-12y)	1771 (10.2)	192 (5.8)	88 (4.9)	214 (6.2)	358 (12.3)	566 (16.6)	353 (15.2)
Adolescence (12-18y)	119 (0.7)	15 (0.5)	8 (0.4)	20 (0.6)	24 (0.8)	33 (1.0)	19 (0.8)
p-value	<0.0001	<0.0001	<0.0001	<0.0001	<0.0001	<0.0001	<0.0001
Gender							
Male	9733 (56.7)	1926 (58.7)	1091 (60.1)	1958 (57.0)	1617 (55.7)	1861 (54.5)	1280 (55.0)
Female	7447 (43.3)	1357 (41.3)	723 (39.9)	1479 (43.0)	1286 (44.3)	1555 (45.5)	1047 (45.0)
p-value	0.0646	0.0157	0.0028	0.0477	0.1179	0.2008	0.1573
Diseases							
Pneumonia	10793 (62.8)	1922 (58.5)	1188 (65.5)	2154 (62.7)	1810 (62.3)	2217 (64.9)	1502 (64.5)
Upper respiratory infections	5270 (30.7)	1184 (36.1)	542 (29.9)	1057 (30.8)	854 (29.4)	888 (26.0)	745 (32.0)
Septicemia	203 (1.2)	55 (1.7)	34 (1.9)	46 (1.3)	19 (0.7)	31 (0.9)	18 (0.8)
Others^a^	914 (5.3)	122 (3.7)	50 (2.8)	180 (5.2)	220 (7.6)	280 (8.2)	62 (2.7)
p-value	<0.0001	<0.0001	<0.0001	<0.0001	<0.0001	<0.0001	<0.0001
Season							
Spring	4250 (24.7)	639 (19.5)	62 (3.4)	852 (24.8)	1028 (35.4)	1165 (34.1)	504 (21.7)
Summer	3688 (21.5)	729 (22.2)	201 (11.1)	749 (21.8)	822 (28.3)	696 (20.4)	491 (21.1)
Autumn	4922 (28.6)	955 (29.1)	771 (42.5)	1017 (29.6)	494 (17)	960 (28.1)	725 (31.2)
Winter	4320 (25.1)	960 (29.2)	780 (43)	819 (23.8)	559 (19.3)	595 (17.4)	607 (26.1)
p-value	0.7159	0.2473	<0.0001	0.5939	0.0096	0.0220	0.2953

a Others included bronchitis, asthma, acute bronchiolitis and acute laryngotracheal bronchitis.

### Detection rate of *S. pneumoniae*

17,180 were *S. pneumoniae*

From 2019 to 2024, a total of 99,709 bacterial strains were isolated from various clinical specimens, of which 37,449 were Gram-positive bacteria. Among these, accounting for 17.2% of all clinical isolates and 45.9% of the Gram-positive bacteria. In total, 216 isolates were obtained from the IPD group, and the remaining isolates were from the non-IPD group.

### Distribution of *S. pneumoniae* in various specimens

The detection rate of *S. pneumoniae* was highest in lower respiratory tract specimens (sputum and lavage fluid, n=16,823), representing 97.9% of all isolates. The numbers of isolates detected from venous blood, pus, secretions (mainly from the ears and eyes), and cerebrospinal fluid were 196, 74, 61, and 12, respectively, corresponding to detection rates of 1.1%, 0.4%, 0.4%, and 0.1%. A total of 14 isolates were detected in other specimens (such as joint fluid, bone marrow, pleural effusion, and peritoneal fluid), with a detection rate of 0.1%. The distribution of *S. pneumoniae* in different specimens is shown in **Table 2**.

**Table 2:** The distribution of *S. pneumoniae* in different specimens.

Specimen Type	2019 (N = 3283)		2020 (N = 1814)		2021 (N = 3437)		2022 (N = 2903)		2023 (N = 3416)		2024 (N = 2327)		Total number (N=17180)	
	%	RANK	%	RANK	%	RANK	%	RANK	%	RANK	%	RANK	%	RANK
Respiratory Specimens^a^	3200 (97.5)	1	1767 (97.4)	1	3367 (98.0)	1	2844 (98.0)	1	3361 (98.4)	1	2284 (98.2)	1	16823 (97.9)	1
Blood	54 (1.6)	2	33 (1.8)	2	46 (1.3)	2	18 (0.6)	3	29 (0.8)	2	16 (0.7)	2	196 (1.1)	2
Purulent fluid	7 (0.2)	4	8 (0.4)	3	15 (0.4)	3	21 (0.7)	2	11 (0.3)	4	12 (0.5)	3	74 (0.4)	3
Secretions	16 (0.5)	3	2 (0.1)	5	6 (0.2)	4	16 (0.6)	4	12 (0.4)	3	9 (0.4)	4	61 (0.4)	4
Cerebrospinal Fluid	4 (0.1)	5	4 (0.2)	4	1 (0.0)	6	2 (0.1)	5	0	6	1 (0.0)	6	12 (0.1)	6
Others	2 (0.1)	6	0 (0.0)	6	2 (0.1)	5	2 (0.1)	6	3 (0.1)	5	5 (0.2)	5	14 (0.1)	5

a Respiratory Specimens include Sputum and bronchoalveolar lavage fluid.

### Antimicrobial susceptibility of *S.pneumoniae*

**Figure 1:**
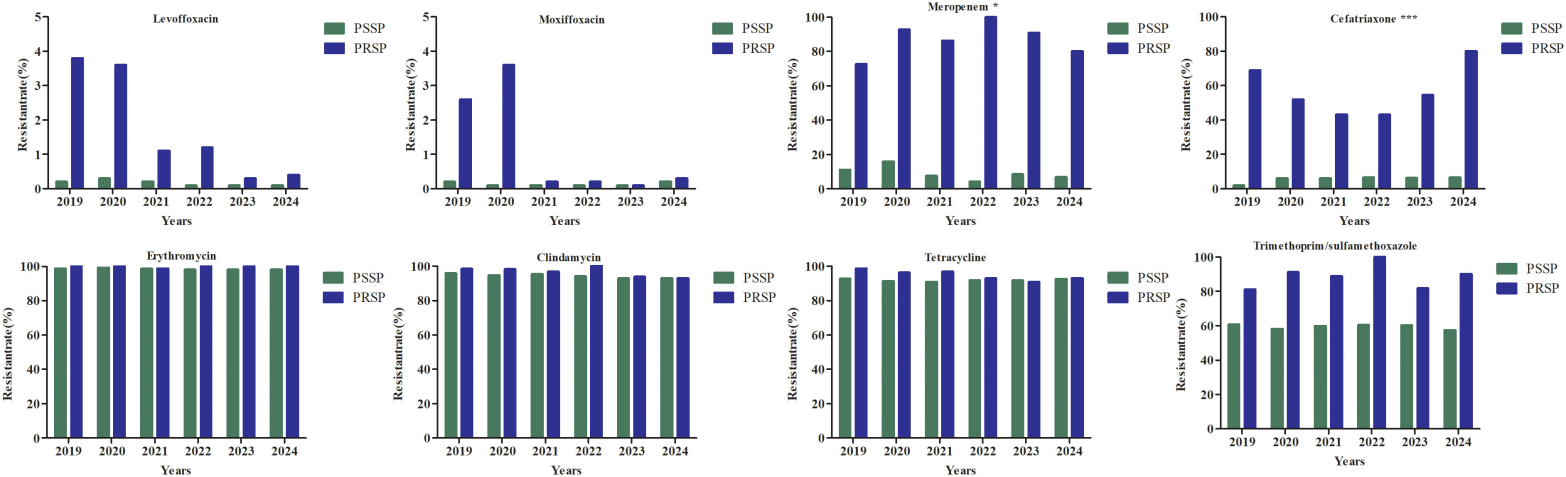
The resistance rate of penicillin-susceptible *S. pneumoniae* and penicillin-resistant *S. pneumoniae.* *P < 0.050, ***P < 0.001.

**Figure 2:**
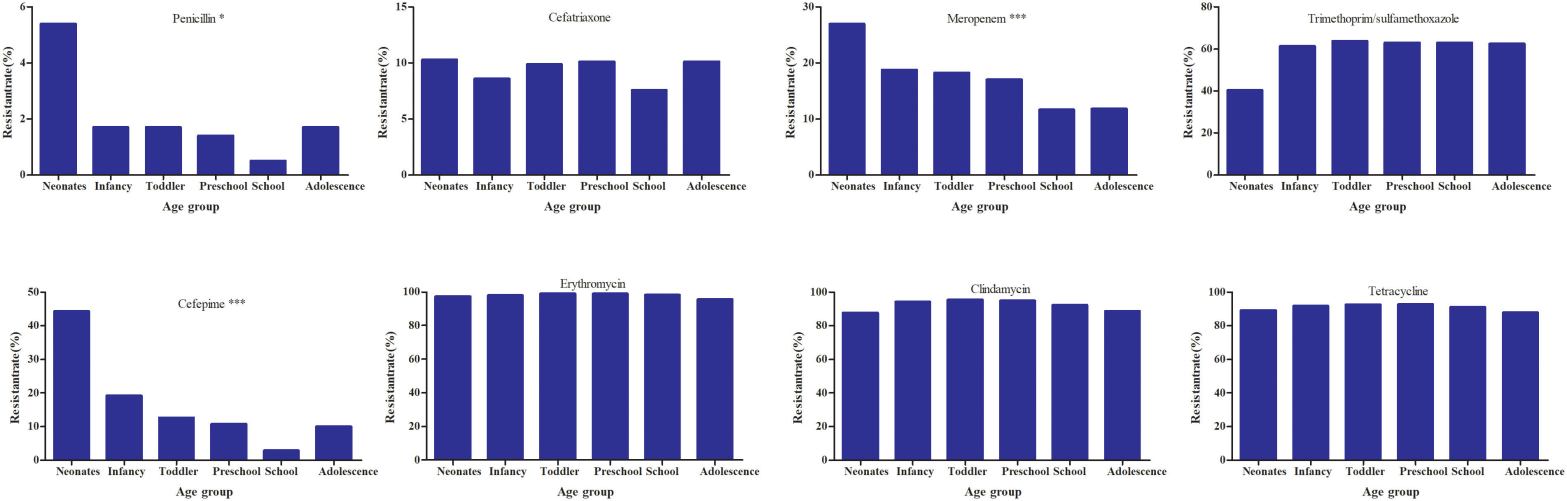
The resistance rate of non-IPD to antibacterial drugs in children of different age groups. The age groups for children refer to the 4th edition of “Child Health Care”,Neonates:≤28days, Infancy:29 days-1 year,Toddler stage:1–3 year,Preschool stage:3–6 year,School age:6–12 year, Adolescence:12-18year. *P < 0.050, ***P < 0.001.

**Figure 3:**
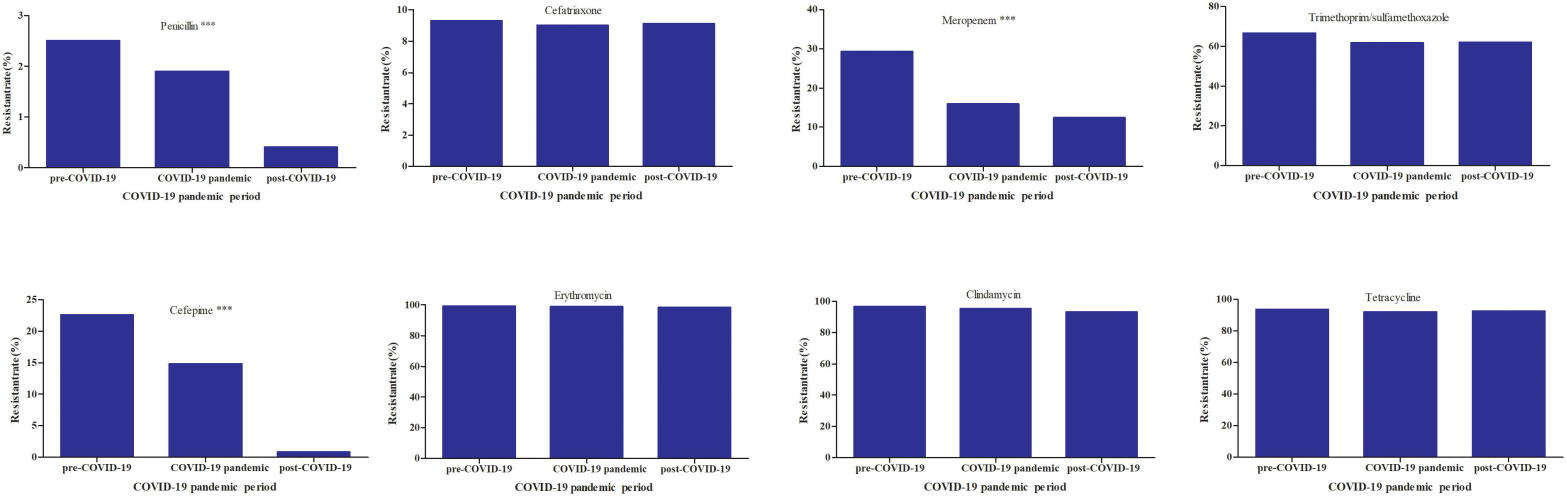
The impact of the COVID-19 pandemic on the antibiotic resistance rate of *S.pneumoniae.* COVID-19 period division:the study period (2019–2024) was divided into the pre-COVID-19 pandemic years (2019), COVID-19 pandemic years (2020–2022), and post-COVID-19 pandemic years (2023–2024). ***P < 0.001.

The resistance rate to moxifloxacin was below 1.0%. Compared with levofloxacin, moxifloxacin usually showed lower MIC_50_ values against *S. pneumoniae* (2 μg/mL vs 0.5 μg/mL). The resistance rates to penicillin, trimethoprim/sulfamethoxazole, erythromycin, clindamycin, cefotaxime, and cefepime showed significant decreasing trends over the six-year period, as shown in **Table 3**. The resistance rates to trimethoprim/sulfamethoxazole,levofloxacin,and moxifloxacin were higher in the non-IPD group than in the IPD group, whereas the resistance rate to chloramphenicol was lower in the non-IPD group. These differences were statistically significant, as shown in **Table 4**. Based on the MIC breakpoints for oral penicillin, the overall proportions of penicillin-susceptible *S. pneumoniae* (PSSP), penicillin-intermediate *S. pneumoniae* (PISP), and penicillin-resistant *S. pneumoniae* (PRSP) isolates were 83.7% (14,196/16,964), 14.8% (2513/16,964), and 1.5% (255/16,964), respectively. Except for vancomycin and linezolid, the resistance rates to all other tested antimicrobial agents were higher in the PRSP group than in the PSSP group (**Figure 1**). In the toddler stage, the resistance rates to trimethoprim/sulfamethoxazole and clindamycin were the highest, whereas in the preschool stage, the resistance rate to erythromycin was highest. The resistance rates to penicillin,cefepime and meropenem showed significant differences among age groups (**Figure 2**). During the COVID-19 pandemic and post-COVID-19 periods, the average annual number of *S. pneumoniae* isolates decreased. Except for chloramphenicol, the resistance rates to all other antibacterial drugs were lower than those before the pandemic(**Figure 3**).

**Table 3:** Changes in the antibiotic resistance rate of non-IPD.

Antibiotics	2019		2020		2021		2022		2023		2024		Average	P-value
	Resistant %	MI_C50_	Resistant %	MI_C50_	Resistant %	MI_C50_	Resistant %	MI_C50_	Resistant %	MI_C50_	Resistant %	MI_C50_	Resistant %	
**penicillin**	**2.5**	**2**	**3.2**	**2**	**2.8**	**2**	**0.1**	**1**	**0.3**	**2**	**0.4**	**2**	**1.5**	**<0.0001**
**Trimethoprim/sulfamethoxazole**	**66.7**	**4**	**61.7**	**4**	**61.7**	**4**	**61.9**	**4**	**62.9**	**4**	**61**	**4**	**62.9**	**0.0002**
levofloxacin	0.2	2	0.2	2	0.2	2	0	2	0.1	2	0.2	2	0.1	**0.2599**
**moxifloxacin**	**0.2**	**0.5**	**0.2**	**0.5**	**0.1**	**0.5**	**0.1**	**0.5**	**0**	**0.5**	**0.1**	**0.5**	**0.1**	**0.0127**
Vancomycin	0	0.5	0	0.5	0	0.5	0	0.5	0	0.5	0	0.5	0	—
Linezolid	0	2	0	2	0	2	0	2	0	2	0	2	0	—
**Erythromycin**	**99.2**	**8**	**99.4**	**8**	**98.7**	**8**	**98.2**	**8**	**98.3**	**8**	**98.3**	**8**	**98.6**	**<0.0001**
**Clindamycin**	**96.5**	**2**	**95.8**	**2**	**95.6**	**2**	**94.3**	**2**	**93.1**	**2**	**93.3**	**2**	**94.7**	**<0.0001**
Tetracycline	93.6	16	92.3	16	91.6	16	91.9	16	92.2	16	92.5	16	92.4	0.1420
**Chloramphenicol**	**8.9**	**4**	**9.3**	**4**	**11**	**4**	**15.4**	**4**	**13.6**	**4**	**11.3**	**4**	**11.2**	**<0.0001**
Rifampin	0.3	1	0.3	0.5	0.2	0.5	0	0.5	0.1	0.5	0.1	0.5	0.1	0.0584
ceftriaxone	9.3	0.5	10.6	1	9.1	0.5	8.1	0.5	9.3	0.5	11	1	9.4	0.4345
**Cefotaxime**	**17.1**	**1**	**12.7**	**1**	**12.4**	**1**	**2.2**	**1**	**2.9**	**1**	**8.9**	**1**	**10.2**	**<0.0001**
**Cefepime**	**22.8**	**2**	**24.1**	**2**	**16.1**	**2**	**1.6**	**1**	**0.9**	**1**	**0.5**	**1**	**12**	**<0.0001**
**Meropenem**	**29.3**	**0.5**	**30.5**	**0.5**	**15.9**	**0.5**	**6.9**	**0.25**	**13**	**0.5**	**11.5**	**0.5**	**17.3**	**<0.0001**

Bold indicates that the difference is statistically signiﬁcant.

**Table 4:** The resistance rate of IPD and non-IPD to antibacterial drugs.

Antibiotics	IPD (N = 216)		Non-IPD(N = 16964)		P-value
	Resistant %	MI_C50_	Resistant %	MI_C50_	
penicillin	2.8	2	1.5	2	0.1256
**Trimethoprim/sulfamethoxazole**	**53.2**	**4**	**62.9**	**4**	**0.0035**
levofloxacin	0	2	0.1	2	0.6416
moxifloxacin	0	0.5	0.1	0.5	0.6416
Vancomycin	0	0.5	0	0.5	—
Linezolid	0	2	0	2	—
Erythromycin	99.0	8	98.6	8	0.5569
Clindamycin	95.4	2	94.7	2	0.6621
**Tetracycline**	**87.0**	**16**	**92.4**	**16**	**0.0032**
**Chloramphenicol**	**18.2**	**4**	**11.2**	**4**	**0.0016**
**Rifampin**	**0.7**	**0.5**	**0.1**	**0.5**	**0.0003**
ceftriaxone	7.4	0.5	9.4	0.5	0.3176
Cefotaxime	11.4	1	10.2	1	0.5070
Cefepime	11.8	2	12.0	1	0.8475
Meropenem	17.6	0.5	17.3	0.5	0.9105

Bold indicates that the difference is statistically signiﬁcant.

### Serotype distribution and vaccine coverage

**Figure 4:**
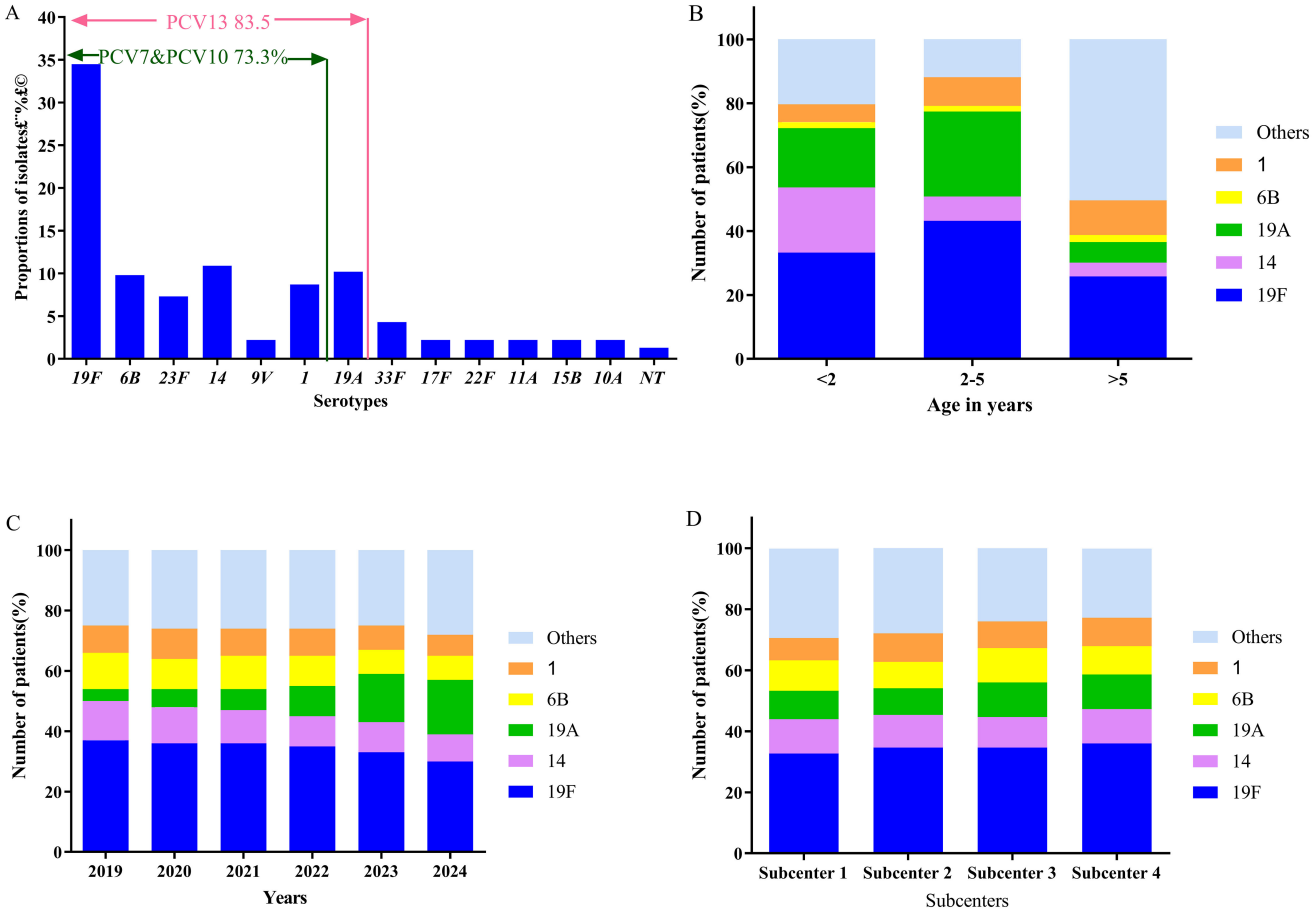
The serotype distribution of the *S. pneumoniae* isolates. **(A)** Serotype distribution and coverage rate of PCVs. **(B)** Serotype distribution in different ages. **(C)** Serotype distribution in different years. **(D)** Serotype distribution in different subcenters. NT indicates non-typeable serotypes.

Except for 8 (1.3%) non-typeable isolates, 13 serotypes were identified among the remaining isolates. The top five serotypes accounted for 74.1% of isolates, namely 19F (n=207, 34.5%), 14 (n=65, 10.9%), 19A (n=61, 10.2%), 6B (n=59, 9.8%), and 1 (n=52, 8.7%). According to these results, PCV7 covered 388 isolates (64.6%), PCV10 covered 440 isolates (73.3%), and PCV13 covered 501 isolates (83.5%)`(**Figure 4A**). The detection rate of serotype 14 was the highest in children under 2 years of age. The highest detection rates of serotypes 19F and 19A were observed in children aged 2–5 years, whereas serotypes 6B and 1 were most frequently detected in children older than 5 years. There were statistically significant differences in the detection rates of serotypes 19F, 14, and 19A among age groups (**Figure 4B**). The detection rate of serotype 19A showed a significant increasing trend over the study period (P = 0.0035) (**Figure 4C**), whereas the annual detection rates of other major serotypes did not differ significantly. Serotype 19F was the most common serotype in all four subcenters`, and there were no significant differences in serotype distribution among subcenters (**Figure 4D**).

## Discussion

*S. pneumoniae* is a major bacterial pathogen responsible for high pneumonia-related morbidity and mortality in children under five years of age. In 2016, the global burden of pneumococcal infections was estimated at 1.18 million deaths (Collaborators, 2018). In analysis of mortality associated with 33 bacterial genera in 2019, pneumococci were associated with the highest number of deaths in young children (Collaborators, 2022). *S. pneumoniae* has shown an increasing trend in resistance to most antibiotics, which hinders the effectiveness of treatment. This is the first multicenter study on epidemiology, changing resistance trends, and serotype distribution of *S. pneumoniae* in children conducted in Chongqing region, based on 17,180 *S. pneumoniae* isolates collected from 2019 to 2024.

In our study, *S. pneumoniae* accounted for 17.2% of all clinical isolates from children during the study period, which is similar to the 15.0% reported by Langhuan Lei (Lei and Wang, 2022). However, this rate was lower than the 30.4% reported in children in Hainan Province, China (Wang et al., 2024) and the 40.6% reported in children in Indonesia (Purwanto et al., 2024), but higher than the 12.3% reported in Shandong Province, China (Yang et al., 2025). Most isolates in our study were obtained from sputum and bronchoalveolar lavage fluid specimens of the lower respiratory tract, suggesting that *S. pneumoniae* is closely associated with respiratory tract infections. Isolates from blood and ear or eye secretions also suggested that *S. pneumoniae* is an important cause of bacteremia and otitis media. Such invasive infections usually have severe clinical manifestations and a poor prognosis.

We found that *S. pneumoniae* was more frequently isolated from infants, toddlers, and preschool-age children, which together accounted for 88.8% of all isolates. This may be due to the fact that the immune systems of infants and young children are not yet fully developed and their resistance to pathogens is relatively poor. At the same time, *S. pneumoniae* infection is closely related to the host’s immune status. Therefore, enhancing the immunity of infants and young children is an effective strategy to prevent *S. pneumoniae* infection. Pneumococcal vaccines can not only reduce the incidence and mortality of pneumococcal pneumonia, but may also reduce resistance to antibacterial drugs. Timely pneumococcal vaccination in early childhood remains the most effective measure to prevent pneumococcal pneumonia.

In our study, PSSP, PISP, and PRSP accounted for 83.7%, 14.8%, and 1.5% of isolates, respectively, which differed from the 41.4%, 17.8%, and 40.8% reported in Beijing, China (Zhao et al., 2022). The resistance rates of PRSP to common antibacterial drugs were higher than those of PSSP, which is consistent with a previous report (Zhao et al., 2022). Antibacterial drug therapy is the preferred method for the clinical treatment of *S. pneumoniae* infection. This study shows that the resistance rates of *S. pneumoniae* to penicillin and cefepime were 1.5% and 12.0%, respectively, which were higher than 0.0% and 8.3% in a previous report (Yan et al., 2024). Furthermore, the resistance rates of *S. pneumoniae* to cefotaxime, ceftriaxone, erythromycin, and clindamycin were 10.2%, 9.4%, 98.6% and 94.7%*, respectively*, which were lower than the 16.7%, 12.5%, 100%, and 100% reported in Southwest China (Yan et al., 2024). The resistance rates to penicillin, cefotaxime, erythromycin and clindamycin in our study were higher than the 0.3%,1.3%,85% and 78.2% of those reported in the Central Vietnam study (Wambugu et al., 2023). The resistance rate to chloramphenicol was 11.2%, lower than 32.3% reported by the Central Vietnam study (Wambugu et al., 2023) and higher than the 9.1% reported by Qian Geng (Geng et al., 2014). By contrast, the resistance to erythromycin and clindamycin were similar to the 99.1% and 95.9% reported by Ziyi Yan (Yan et al., 2021) and lower than 99.1% and 98.1% reported by Ge Dai (Geng et al., 2014). The MIC results for erythromycin and clindamycin suggested that the MLS_B_ phenotype was the most prevalent phenotype in China, which is consistent with previous research (Li et al., 2011). Importantly, the data from this study reveal that the penicillin resistance rate of *S. pneumoniae* gradually decreased from 2.5% in 2019 to 0.4% in 2024, showing a year-by-year downward trend. A similar trend has also been observed in the United States (Suaya et al., 2020), which may be related to the widespread use of the PCV13 vaccine and reduced use of penicillin.

We also observed that the average annual detection rate of *S. pneumoniae* decreased in the post-COVID-19 period. Meanwhile, except for chloramphenicol, the resistance rates to other antibacterial drugs all decreased. This may be related to the COVID-19 pandemic that began in 2019 and the subsequent lockdown measures implemented to limit viral transmission (for example, online schooling, mask-wearing), which not only reduced the transmission of COVID-19 but also decreased the spread of other microorganisms. At the same time, these measures may also have affected the pathogenicity of *S.pneumoniae* (Amin-Chowdhury et al., 2021). Although the incidence of pneumococcal disease declined substantially during the COVID-2019 pandemic, the mortality rate associated with IPD/COVID-19 co-infection remains extremely high (Mitsi et al., 2022), underscoring the need for continued vigilance Consistent with our results section, resistance rates to trimethoprim/sulfamethoxazole, levofloxacin, and moxifloxacin, were higher among non-IPD isolates than among IPD isolates, whereas the ceftriaxone, cefotaxime and clindamycin resistance were lower in non-IPD isolates, partially differing from the literature reported (Yan et al., 2021). The resistance rates of *S. pneumoniae* to fluoroquinolone drugs levofloxacin and moxifloxacin remained in a low resistance state (less than 1%) for six years, which was basically consistent with the levofloxacin resistance rate of less than 2% reported in the Canadian study (Patel et al., 2011). Although levofloxacin has the advantages of high sensitivity, strong activity and broad antibacterial spectrum against drug-resistant pneumococcus and can be used for clinical empirical treatment, it has an impact on bone development, which limits its common use in children. No resistance of *S. pneumoniae* to vancomycin and linezolid has been found to date. However, vancomycin has nephrotoxicity and linezolid has a myelosuppressive effect. Therefore, these agents are generally used less frequently in clinical practice.

The predominant serotypes identified in this study were 19F, 14, 19A, 6B and 1, which together accounted for 74.1% of the isolates. In Suzhou, the main prevalent serotypes included 19F, 19A, 6B, 23F, and 6A (Dai et al., 2023), whereas studies from Southwest China (19F, 19A, 6B, 6A, and 14) (Yan et al., 2021), Shenzhen (23A, 15A, 6E and 34) (Shi et al., 2023), Central Vietnam(6A/B, 19F and 23F) (Wambugu et al., 2023),and Northern Beijing (19F, 19A, 23F, 14, 6A) (Wang et al., 2020) have reported different serotype patterns. These findings highlight substantial regional variation in *S. pneumoniae* serotype distribution, which may be related to local antibiotic usage habits, climate, etc. In our study, the estimated serotype coverage rates of PCV7, PCV10 and PCV13 were 64.6%, 73.3% and 83.5%, respectively. This finding was lower than that of the study in Shanghai, China (PCV13 89.9%) (Geng et al., 2014), but higher than the 62% reported among adults in Beijing (Zhao et al., 2022). The increased coverage of PCV13 in our data was mainly due to the high prevalence of serotype 19A, consistent with other countries (Croucher et al., 2011). In 2001, PCV7 was approved in Europe and was introduced into the national immunization programs of many European countries from 2006 to 2008. PCV10 and PCV13 were adopted to replace PCV7 from 2009 to 2011.This has led to changes in the prevalent serotypes in the European region. In countries using PCV13, serotypes 24F, 22F, 8 and 15A became predominant, while in countries using PCV10, serotypes 19A and 3 remained common. This indicates the importance of continuous monitoring of serotypes dynamics and their impact on vaccine effectiveness. Following the introduction of PCVs, vaccine serotypes decreased and serotype replacement has been observed worldwide, especially for serotype 19A (Croucher et al., 2011). Our research also observed this phenomenon. Although PCV7 has been available in China since 2008, vaccine uptake remains very low. Studies have reported that approximately 86.0% of children with respiratory tract infections had used one or more antibiotics before admission. In addition, a large number of domestic and foreign floating population may be an important factor leading to serotype replacement in Chongqing area. Notably, PCV7 used in our country does not include serotype 19A; thus, as PCV7 coverage increases, serotype 19A may become more prevalent. Therefore, it is recommended to use the PCV13 vaccine with a higher coverage rate to prevent *S. pneumoniae* infection.

In conclusion, this multicenter study provides valuable data on the prevalence, antimicrobial susceptibility, and serotype distribution of *S. pneumoniae* among children in the Chongqing region. Our findings confirm the excellent *in vitro* activity of vancomycin, linezolid, levofloxacin and moxifloxacin against *S. pneumoniae*, but also highlight the high resistance to macrolide drugs. The most prevalent serotypes of *S. pneumoniae* infection in children in Chongqing region are 19F, 14, 19A, 6B and 1. Considering the relatively high coverage rate of PCV13 and the worrying rate of antibiotic resistance, broader use of PCV13 may be a promising preventive strategy to control the increasing trend of clonal spread in Chongqing region of China.

## Limitations

It is important to note that this was a retrospective four-center study. The provided data cannot represent the overall situation of epidemiology and serotype prevalence of *S. pneumoniae* in China. However, considering the increasing vaccination rates year by year, it can serve as a basis for predicting future trends. In the future, we still need to expand the sample size in order to obtain more accurate and reliable research conclusions. This is because 97.9% of the S. pneumoniae isolates in our study were obtained from sputum and bronchoalveolar lavage fluid, with only 1.3% being invasive strains. To address these limitations, we plan to extend our work by including samples from other parts of the country and invasive strains to gain a more comprehensive understanding of the drug resistance patterns. This could potentially provide valuable insights into the emergence of multidrug-resistant non-vaccine type serotypes and guide the development of effective prevention and treatment strategies.

## Data Availability

The original contributions presented in the study are included in the article/supplementary material. Further inquiries can be directed to the corresponding author.

## References

[B1] Amin-Chowdhury Z. Aiano F. Mensah A. Sheppard C. L. Litt D. Fry N. K. . (2021). Impact of the coronavirus disease 2019 (COVID-19) pandemic on invasive pneumococcal disease and risk of pneumococcal coinfection with severe acute respiratory syndrome coronavirus 2 (SARS-coV-2): prospective national cohort study, england. Clin. Infect. Dis. 72, e65–e75. doi: 10.1093/cid/ciaa1728, PMID: 33196783 PMC7717180

[B2] Chan J. Nguyen C. D. Dunne E. M. Kim Mulholland E. Mungun T. Pomat W. S. . (2019). Using pneumococcal carriage studies to monitor vaccine impact in low- and middle-income countries. Vaccine 37, 6299–6309. doi: 10.1016/j.vaccine.2019.08.073, PMID: 31500968

[B3] Clinical and Laboratory Standards Institute (CLSI) (2024). Performance standards for antimicrobial susceptibility testing. 34th (Wayne, PA: CLSI). CLSI supplement M100.

[B4] Collaborators GBDLRI. (2018). Estimates of the global, regional, and national morbidity, mortality, and aetiologies of lower respiratory infections in 195 countries, 1990-2016: a systematic analysis for the Global Burden of Disease Study 2016. Lancet Infect. Dis. 18, 1191–1210. doi: 10.1016/S1473-3099(18)30310-4, PMID: 30243584 PMC6202443

[B5] Collaborators GBDAR. (2022). Global mortality associated with 33 bacterial pathogens in 2019: a systematic analysis for the Global Burden of Disease Study 2019. Lancet 400, 2221–2248. doi: 10.1016/S0140-6736(22)02185-7, PMID: 36423648 PMC9763654

[B6] Croucher N. J. Harris S. R. Fraser C. Quail M. A. Burton J. van der Linden M. . (2011). Rapid pneumococcal evolution in response to clinical interventions. Science 331, 430–434. doi: 10.1126/science.1198545, PMID: 21273480 PMC3648787

[B7] Dai G. Wang T. He Y. Jiang W. Sun H. Chen Z. . (2023). Antimicrobial susceptibility and serotype distribution of Streptococcus pneumoniae isolates among children in Suzhou, China. Transl. Pediatr. 12, 2203–2212. doi: 10.21037/tp-23-547, PMID: 38197098 PMC10772826

[B8] Ganaie F. Saad J. S. McGee L. van Tonder A. J. Bentley S. D. Lo S. W. . (2020). A new pneumococcal capsule type, 10D, is the 100th serotype and has a large cps fragment from an oral streptococcus. mBio 11, e00937–20. doi: 10.1128/mBio.00937-20, PMID: 32430472 PMC7240158

[B9] Geng Q. Zhang T. Ding Y. Tao Y. Lin Y. Wang Y. . (2014). Molecular characterization and antimicrobial susceptibility of Streptococcus pneumoniae isolated from children hospitalized with respiratory infections in Suzhou, China. PloS One 9, e93752. doi: 10.1371/journal.pone.0093752, PMID: 24710108 PMC3977860

[B10] Johnson H. L. Deloria-Knoll M. Levine O. S. Stoszek S. K. Freimanis Hance L. Reithinger R. . (2010). Systematic evaluation of serotypes causing invasive pneumococcal disease among children under five: the pneumococcal global serotype project. PloS Med. 7, e1000348. doi: 10.1371/journal.pmed.1000348, PMID: 20957191 PMC2950132

[B11] Kawaguchiya M. Urushibara N. Aung M. S. Ohashi N. Tsutida S. Kurashita K. . (2024). Serotype distribution and antimicrobial resistance of Streptococcus pneumoniae isolated from children in Japan, 2023. New Microbes New Infect. 62, 101513. doi: 10.1016/j.nmni.2024.101513, PMID: 39507501 PMC11539348

[B12] Lei L. Wang X. (2022). Determining the frequency of Streptococcus pneumoniae carriers and its microbial resistance in children. Cell Mol. Biol. (Noisy-le-grand). 68, 203–207. doi: 10.14715/cmb/2022.68.2.29, PMID: 35869729

[B13] Li L. Ma J. Yu Z. Li M. Zhang W. Sun H. (2023). Epidemiological characteristics and antibiotic resistance mechanisms of Streptococcus pneumoniae: An updated review. Microbiol. Res. 266, 127221. doi: 10.1016/j.micres.2022.127221, PMID: 36244081

[B14] Li Y. Tomita H. Lv Y. Liu J. Xue F. Zheng B. . (2011). Molecular characterization of erm(B)- and mef(E)-mediated erythromycin-resistant Streptococcus pneumoniae in China and complete DNA sequence of Tn2010. J. Appl. Microbiol. 110, 254–265. doi: 10.1111/j.1365-2672.2010.04875.x, PMID: 20961364

[B15] Lo S. W. Mellor K. Cohen R. Alonso A. R. Belman S. Kumar N. . (2022). Emergence of a multidrug-resistant and virulent Streptococcus pneumoniae lineage mediates serotype replacement after PCV13: an international whole-genome sequencing study. Lancet Microbe 3, e735–e743. doi: 10.1016/S2666-5247(22)00158-6, PMID: 35985351 PMC9519462

[B16] Lyu S. Shi W. Dong F. Xu B. P. Liu G. Wang Q. . (2024). Serotype distribution and antimicrobial resistance of pediatric Streptococcus pneumoniae isolated from inpatients and outpatients at Beijing Children’s Hospital. Braz. J. Infect. Dis. 28, 103734. doi: 10.1016/j.bjid.2024.103734, PMID: 38471654 PMC11004498

[B17] Mai W. Liu Y. Meng Q. Xu J. Wu J. (2023). Bacterial epidemiology and antimicrobial resistance profiles of respiratory specimens of children with pneumonia in hainan, China. Infect. Drug Resist. 16, 249–261. doi: 10.2147/idr.S397513, PMID: 36660346 PMC9842527

[B18] Men W. Dong Q. Shi W. Yao K. (2020). Serotype distribution and antimicrobial resistance patterns of invasive pneumococcal disease isolates from children in mainland China-a systematic review. Braz. J. Microbiol. 51, 665–672. doi: 10.1007/s42770-019-00198-9, PMID: 31797324 PMC7203282

[B19] Mitsi E. Reiné J. Urban B. C. Solórzano C. Nikolaou E. Hyder-Wright A. D. . (2022). Streptococcus pneumoniae colonization associates with impaired adaptive immune responses against SARS-CoV-2. J. Clin. Invest. 132, e157124. doi: 10.1172/jci157124, PMID: 35139037 PMC8970672

[B20] Namkoong H. Ishii M. Funatsu Y. Kimizuka Y. Yagi K. Asami T. . (2016). Theory and strategy for Pneumococcal vaccines in the elderly. Hum. Vaccin Immunother. 12, 336–343. doi: 10.1080/21645515.2015.1075678, PMID: 26406267 PMC5049722

[B21] Narciso A. R. Dookie R. Nannapaneni P. Normark S. Henriques-Normark B. (2025). Streptococcus pneumoniae epidemiology, pathogenesis and control. Nat. Rev. Microbiol. 23, 256–271. doi: 10.1038/s41579-024-01116-z, PMID: 39506137

[B22] Patel S. N. McGeer A. Melano R. Tyrrell G. J. Green K. Pillai D. R. . (2011). Susceptibility of Streptococcus pneumoniae to fluoroquinolones in Canada. Antimicrob. Agents Chemother. 55, 3703–3708. doi: 10.1128/AAC.00237-11, PMID: 21628545 PMC3147653

[B23] Pimenta F. Moiane B. Gertz R. E. Jr. Chochua S. Snippes Vagnone P. M. Lynfield R. . (2021). New pneumococcal serotype 15D. J. Clin. Microbiol. 59, e00329–21. doi: 10.1128/JCM.00329-21, PMID: 33658265 PMC8091843

[B24] Purwanto D. S. Khoeri M. M. Tafroji W. Margaretha Kaligis S. H. Wilar R. Johnson Kepel B. . (2024). Nasopharyngeal carriage rate, serotype distribution, and antimicrobial profiles of Streptococcus pneumoniae among patients with acute respiratory tract infection in Manado, North Sulawesi, Indonesia. Access Microbiol. 6 000703.v4. doi: 10.1099/acmi.0.000703.v4, PMID: 38725588 PMC11077345

[B25] Shi X. Patil S. Wang Q. Liu Z. Zhu C. Wang H. . (2023). Prevalence and resistance characteristics of multidrug-resistant Streptococcus pneumoniae isolated from the respiratory tracts of hospitalized children in Shenzhen, China. Front. Cell Infect. Microbiol. 13. doi: 10.3389/fcimb.2023.1332472, PMID: 38268793 PMC10806184

[B26] Suaya J. A. Mendes R. E. Sings H. L. Arguedas A. Reinert R. R. Jodar L. . (2020). Streptococcus pneumoniae serotype distribution and antimicrobial nonsusceptibility trends among adults with pneumonia in the United States, 2009–2017. J. Infect. 81, 557–566. doi: 10.1016/j.jinf.2020.07.035, PMID: 32739491

[B27] Tran-Quang K. Nguyen-Thi-Dieu T. Tran-Do H. Pham-Hung V. Nguyen-Vu T. Tran-Xuan B. . (2023). Antibiotic resistance of Streptococcus pneumoniae in Vietnamese children with severe pneumonia: a cross-sectional study. Front. Public Health 11. doi: 10.3389/fpubh.2023.1110903, PMID: 37383272 PMC10294427

[B28] Wambugu P. Shah M. M. Nguyen H. A. Le K. A. Le H. H. Vo H. M. . (2023). Molecular epidemiology of streptococcus pneumoniae detected in hospitalized pediatric acute respiratory infection cases in central Vietnam. Pathogens. 12, 943 doi: 10.3390/pathogens12070943, PMID: 37513790 PMC10385502

[B29] Wang J. Qiu L. Bai S. Zhao W. Zhang A. Li J. . (2024). Prevalence and serotype distribution of nasopharyngeal carriage of Streptococcus pneumoniae among healthy children under 5 years of age in Hainan Province, China. Infect. Dis. Poverty. 13, 7. doi: 10.1186/s40249-024-01175-7, PMID: 38238873 PMC10797996

[B30] Wang Q. Shi W. Li Y. Gao W. Yuan L. Dong F. . (2020). Serotype distribution of Streptococcus pneumoniae isolated from children hospitalized in Beijing children’s hospital (2013-2019). Vaccine 38, 7858–7864. doi: 10.1016/j.vaccine.2020.10.005, PMID: 33164807

[B31] Wang J. Wu Q. S. Lu J. Ni Y. H. Zhou F. (2021). Low vaccination coverage of pneumococcal conjugate vaccines (PCVs) in Shanghai, China: A database analysis based on birth cohorts from 2012 to 2020. Vaccine 39, 6189–6194. doi: 10.1016/j.vaccine.2021.09.011, PMID: 34538698

[B32] Yan Z. Cui Y. Huang X. Lei S. Zhou W. Tong W. . (2021). Molecular characterization based on whole-genome sequencing of streptococcus pneumoniae in children living in southwest China during 2017-2019. Front. Cell Infect. Microbiol. 11. doi: 10.3389/fcimb.2021.726740, PMID: 34796125 PMC8593041

[B33] Yan Z. Miao C. Liu L. Fu Y. Liu X. Li H. . (2024). Antibiotic susceptibility testing and molecular characterization based on whole-genome sequencing of Streptococcus pneumoniae isolates from pediatric infections at the National Regional Medical Center of Southwest China during the COVID-19 pandemic. Front. Public Health 12. doi: 10.3389/fpubh.2024.1490401, PMID: 39720806 PMC11666559

[B34] Yang S. Wang M. Wang S. (2025). Shandong provincial microbiome research center children B, fungal resistance monitoring research N. Bacterial epidemiology and antimicrobial resistance in children in shandong province, China, 2017-2022: A multicentre retrospective study. Infect. Drug Resist. 18, 2823–2836. doi: 10.2147/IDR.S511161, PMID: 40474961 PMC12139632

[B35] Zainel A. Mitchell H. Sadarangani M. (2021). Bacterial meningitis in children: neurological complications, associated risk factors, and prevention. Microorganisms. 9, 535 doi: 10.3390/microorganisms9030535, PMID: 33807653 PMC8001510

[B36] Zeng Y. Song Y. Cui L. Wu Q. Wang C. Coelho A. C. . (2023). Phylogenomic insights into evolutionary trajectories of multidrug resistant S. pneumoniae CC271 over a period of 14 years in China. Genome Med. 15, 46. doi: 10.1186/s13073-023-01200-8, PMID: 37403170 PMC10318735

[B37] Zhao C. Xie Y. Zhang F. Wang Z. Yang S. Wang Q. . (2020). Investigation of antibiotic resistance, serotype distribution, and genetic characteristics of 164 invasive streptococcus pneumoniae from north China between april 2016 and october 2017. Infect. Drug Resist. 13, 2117–2128. doi: 10.2147/idr.S256663, PMID: 32753907 PMC7342493

[B38] Zhao C. Yang S. Zhang F. Wang Z. Zhang Y. Wang X. . (2022). Antimicrobial resistance trends of the most common causative pathogens associated with community-acquired respiratory infections in China: 2009-2018. Infect. Drug Resist. 15, 5069–5083. doi: 10.2147/idr.S374805, PMID: 36071818 PMC9443291

[B39] Zhou X. Liu J. Zhang Z. Cui B. Wang Y. Zhang Y. . (2022). Characterization of Streptococcus pneumoniae Macrolide Resistance and Its Mechanism in Northeast China over a 20-Year Period. Microbiol. Spectr. 10, e0054622. doi: 10.1128/spectrum.00546-22, PMID: 35938873 PMC9602527

